# Nutrient intake, dietary patterns, and anthropometric variables of children with ADHD in comparison to healthy controls: a case-control study

**DOI:** 10.1186/s12887-022-03123-6

**Published:** 2022-01-29

**Authors:** Habibeh Salvat, Mehriar Nader Mohammadi, Parviz Molavi, Seyed Ali Mostafavi, Reza Rostami, Mohammad Ali Salehinejad

**Affiliations:** 1 Department of Psychiatry, Fatemi Hospital, School of Medicine, Ardabil University of Medical Sciences, Ardabil, Iran; 2 Department of Psychiatry, Tehran University of Medical Sciences, Tehran, Iran; 3 Department of Psychology, University of Tehran, Tehran, Iran; 4 Department of Psychology and Neurosciences, Leibniz Research Centre for Working Environment and Human Factors, Dortmund, Germany

**Keywords:** Attention-Deficit Hyperactivity Disorder (ADHD), lifestyle, Anthropometry, Nutrition

## Abstract

**Background:**

Poor health behaviors and variables are recently more documented in attention-deficit hyperactivity disorder (ADHD) lifestyle which might be relevant to the pathophysiology of this disorder. The objective of this case-control study was to assess the nutrient intake, dietary patterns, and anthropometric variables in children with ADHD compared to normal peers.

**Method:**

One hundred children diagnosed with ADHD were included and compared to 100 healthy, sex-matched normal children as the control group. Anthropometric indices, macronutrients, and micronutrients were measured and compared in both groups.

**Results:**

ADHD children were significantly consuming more simple sugars, tea, ready-made meals but less protein, vitamin B1, vitamin B2, vitamin C, zinc and calcium compared to the control group. The body mass index (BMI) and waist circumference of children with ADHD were significantly higher and were related to the severity and type of the disease.

**Conclusion:**

Unhealthy eating behavior is more frequent in children with ADHD, compared to normal children which might warrant lifestyle intervention in this disorder.

## Background

Attention-deficit and Hyperactivity Disorder (ADHD) is a common neurodevelopmental disorder of childhood and adolescence [1] marked with core symptoms of inattention and hyperactivity [2] and cognitive dysfunctions (e.g., working memory, inhibition control) [3, 4] with a well-documented underlying pathophysiology in the brain [5]. There is a consensus that the etiology of ADHD is heterogeneous and results from a complex interaction of pathophysiological and neurochemical systems with genetic phenotype. Other factors that contribute to the onset or sustainability of ADHD symptoms include psychosocial, environmental, and dietary factors [6–8]. The symptoms of inattention, impulsivity, and poor planning are suggested to contribute to poor health behaviors in ADHD patients [9]. Considering such diverse etiological factors, alternative or nonmedical treatments have been also proposed for the treatment or alleviation of ADHD symptoms including several kinds of dietary interventions [10]. In this line, however, there is still an urgent need for studies investigating dietary-related factors between ADHD children and their typically developing peers.

One factor related to dietary behavior is anthropometric indices. The word anthropometry consists of two parts: the *anthropus* means the human and the *metric* means measuring and is referred to as the measurement of human physical indices. One of the aims of anthropometric measurements is to examine the human body size and shape. In the developing population including children and adolescents, anthropometric measurements are applied as variables of growth and development [11]. Other anthropometric variables including body weight, height, body mass index (BMI), skinfold thickness, peripheral measurements and measuring fat mass, and muscle mass are usually used for nutritional evaluations [12].

Some of these anthropometric variables are investigated in children with ADHD. A study in this respect investigated the changes in body and hormone levels in children with ADHD and found that several variables are either lower or higher in children with ADHD when compared to typically-developing controls [13, 14]. In another study, several differences in nutritional patterns were observed between children with ADHD and healthy controls. These differences were “not eating” breakfast, having a history of food allergies which contributed to ADHD symptoms severity, and higher intake of daily sugar in children with more severe ADHD symptoms [15]. Another related study reported a significantly lower weight and height of children with ADHD in comparison with their peers without ADHD [16]. In contrast, another study investigated obesity and overweight of ADHD children and found that 10–17 years old boys and 10–17 years old girls with ADHD were significantly overweight than those in the control group [17]. ADHD as a potential risk factor for obesity and overweight is supported in another study that assessed the prevalence of obesity and overweight in children with ADHD [18].

The association of dietary behavior and ADHD symptoms is important for the well-being, general health, and development of children with ADHD, however, a limited number of studies exist in this respect. Moreover, although the above-mentioned studies provide some insight about nutrients and anthropometric variables in ADHD, no comprehensive investigation of variables is available. Finally, the mixed results from previous studies warrant further investigation in carefully-controlled case studies. Accordingly in the present study, we aimed to assess the dietary patterns, nutrient intake, and anthropometric variables in ADHD children as compared with normal children. Additionally, we assessed the anthropometric variables with regard to ADHD subtype and severity of symptoms.

## Methods

### Study design population

This study adopted a case-control design and performed on children aged 5–13 years old with and without ADHD. Sample size was determined based on previous similar works and using the Cochran formula and 200 children, including 100 children with ADHD (case group) and 100 typically developing controls (control group) were recruited. To reduce the confounding factors, two groups were matched for age and sex. Considering that the sample was selected in Ardabil, all the children present in the study were Azeri and of the same race. Inclusion criteria for the ADHD group were (1) meeting Diagnostic and Statistical Manual of Mental Disorders criteria (DSM-5) [2] for ADHD by consensus of the two experienced child psychiatrists, (2) no history of confounding medical, neurological, and psychiatric conditions, (3) and parent’s scoring on the Conners Rating Scale [19]. Typically developing controls underwent the same evaluation and diagnostic procedure.

### Ethics

All ethical requirements according to the declaration of Helsinki were met. The study protocol was described to parents and their children. We obtained the written informed consent form parents and oral assent from children before participation in the study. Ethical approval was obtained from the ethics committee review board of the Ardabil University of Medical Sciences (ethics code: IR.ARUMS.REC.1395.81).

### Study procedure

Participants in the ADHD group were selected from the patients referred to the Fatemi Hospital of the correspondent university and healthy controls were age and sex-matched from kindergartens and schools during the year 2018. In both groups, children were screened for ADHD by a professional child psychiatrist according to the clinical interview based on the DSM-5 diagnostic criteria. The healthy control group was also interviewed for ruling out ADHD and other neurodevelopmental disorders. The severity of the disorder was furthermore assessed by the Conners questionnaire (parent form) [19]. Then dietary intake and anthropometric variables were assessed in both groups.

### Anthropometric assessment

In this study, anthropometric variableswere measured by a trained physician. Anthropometric parameters were measured in the standard positions. Bodyweight was measured on a calibrated digital scale, to the nearest 0.1 Kg, for this purpose the subjects wore minimal clothes without shoes. Height was measured by an un-stretchable tape on a wall with a straight body position to the nearest 0.1 cm. Waist and arm circumferences were measured by an un-stretchable tape to the nearest 0.1 cm. Body mass index (BMI) was calculated by dividing weight (kg) to height squared (m2).

### Dietary assessment

The children’s nutritional information was collected by the Food Frequency Questionnaires (FFQ) [20]. The guideline for food portion sizes was delivered to parents and they were instructed to fill the FFQ for their children. The parents were asked to sort their children’s average frequency consumption of each food item during the past year according to food portion sizes. Furthermore, a three-day food record was collected from participants. The parents should record all foods and beverages consumed by their children over three randomly selected days (two regular days and one weekend). The gathered data of dietary intake were analyzed by N4 software.

### Statistical analysis

Data analysis was conducted using the SPSS-21 software package. Descriptive statistics including mean and standard deviation were used for data overview and to compare groups (ADHD vs control) in outcome measures (waist circumference, BMI, nutrients intake), independent Student’s t-test was performed. The effect of ADHD subtype (3 values) and symptom severity on each anthropometric variables was also investigated using the univariate analysis of variance (ANOVA). The assumptions of homogeneity of variances and normal distribution were examined with Levene’s test for homogeneity of variances and Shapiro-Wilk test of normality respectively.

## Results

### Data overview

The mean age of the ADHD children in the case group was 8.33 ± 2.10 years and the mean age of the control group was 8.26 ± 2.00 years. 72% of both groups were boys and the rest were girls. There was no significant difference between the groups in these demographic variables. Demographic information and the mean and standard deviation of age, weight, height, BMI, and other anthropometric variables are summarized in Table 1.

**Table 1:** Comparison of different parameters of children in the case and control groups

General category	Parameter	Case group	Control group	Group ***P***-value
Age (Year)	Mean ± SD	8.33 ± 2.08	8.26 ± 2.08	NS
	Min	5	5	
	Max	13	13	
Sex	Male	72 (72%)	72 (72%)	NS
	female	28 (28%)	28 (28%)	
Weight (kg)	Mean	25.69 ± 5.96	25.05 ± 6.60	NS
	Min	14	11.50	
	Max	42.5	48.50	
Height (cm)	Mean	122.68 ± 12.07	123.35 ± 12.43	NS
	Min	92	97	
	Max	152	151	
BMI (kg/Sq. M)	Mean	16.99 ± 2.17	16.16 ± 1.90	0.012^*^
	Min	13.20	11.39	
	Max	28.10	26.04	
Waist Circumference (cm)	Mean	60.96 ± 8.88	58.38 ± 8.69	0.039^*^
	Min	13.2	41	
	Max	28.1	78	
Arm Circumference (cm)	Mean	22.61 ± 4.51	21.51 ± 4.56	NS
	Min	13	12.50	
	Max	40	34.00	

NS: Not significant; * significant at the *p* < 0.05

### Anthropometric variables

In the case group, 28 children were diagnosed with predominantly hyperactive/impulsive type, 11 children with the predominantly inattentive type and 61 children were diagnosed with the combined type of ADHD. Also, in this group according to conners score 49% had mild, 37% had moderate and 14% had severe ADHD symptoms. Table 2 summarized the anthropometric variablesin the children with ADHD subgrouped by type and severity of disease compared with the control group. There was a significant difference between ADHD children and their controls in the mean BMI (*p* = 0.012) and Waist Circumference (*p* = 0.039) (Table 1). The results of ANOVA showed a significant main effect of ADHD type on the BMI and mean waist circumference (Table 2). Post hoc analysis showed a significantly higher mean BMI in the inattentive subtype compared to the hyperactive type. Similarly, the inattentive subtype had a significantly larger mean waist circumference compared to both hyperactive and combined subtypes.

**Table 2:** Anthropometric and type and severity of disease of ADHD in comparison with the control group

General group	Type and severity of disorder	parameters	number	Mean	Standard deviation	F	***p***-values	Pairwise comparisons
**Mean weight (kg)**	Type of Disorder	Control group	100	25.05	6.60	1.342	0.262	na
		Mostly hyperactive	28	24.80	6.25			
		Mostly attention deficit	11	28.90	6.80			
		Combined	61	25.51	5.58			
	The severity of Disorder	Control group	100	25.05	6.60	0.731	0.535	na
		mild	49	25.63	6.55			
		moderate	37	26.44	5.67			
		severe	14	23.89	4.24			
**Mean height (cm)**	Type of Disorder	Control group	100	123.35	12.43	0.827	0.480	na
		Mostly hyperactive	28	122.00	12.67			
		Mostly attention deficit	11	128.00	10.16			
		Combined	61	122.03	12.04			
	The severity of Disorder	Control group	100	123.35	12.43	1.514	0.212	na
		mild	49	121.42	13.19			
		moderate	37	125.81	11.00			
		severe	14	118.78	9.05			
**Mean BMI**	Type of Disorder	Control group	100	16.16	1.90	2.998	0.032^*^	na
		Mostly hyperactive	28	16.38	1.28			
		Mostly attention deficit	11	17.35	1.80			AD>HD
		Combined	61	17.04	2.52			na
	The severity of Disorder	Control group	100	16.16	1.90	2.990	0.032^*^	
		mild	49	17.20	2.82			mild> control, moderate
		moderate	37	16.49	1.18			na
		severe	14	16.84	1.38			
**Mean waist circumference (cm)**	Type of Disorder	Control group	100	803.82	96.73	3.144	0.016^*^	na
		Mostly hyperactive	28	734.46	75.77			
		Mostly attention deficit	11	804.09	82.69			
		Combined	61	780.00	139.79			AD/HD > HD
	The severity of disorder	Control group	100	803.82	96.73	1.670	0.175	na
		mild	49	764.89	145.49			
		moderate	37	777.02	96.64			
		severe	14	768.57	85.40			
**Mean arm circumference (cm)**	Type of disorder	Control group	100	21.51	4.55	2.341	0.075	na
		Mostly hyperactive	28	21.83	4.46			
		Mostly attention deficit	11	25.045	4.17			
		Combined	61	22.53	4.50			
	The severity of disorder	Control group	100	21.51	4.56	1.311	0.272	na
		mild	49	23.06	4.67			
		moderate	37	22.28	4.58			
		severe	14	21.92	3.80			

*na* Not applicable, *AD* Predominantly attentive subtype, *HD* Predominantly hyperactive subtype; Significant at the *p* < 0.05

### Dietary pattern, micro-and micronutrients

**Fig. 1:**
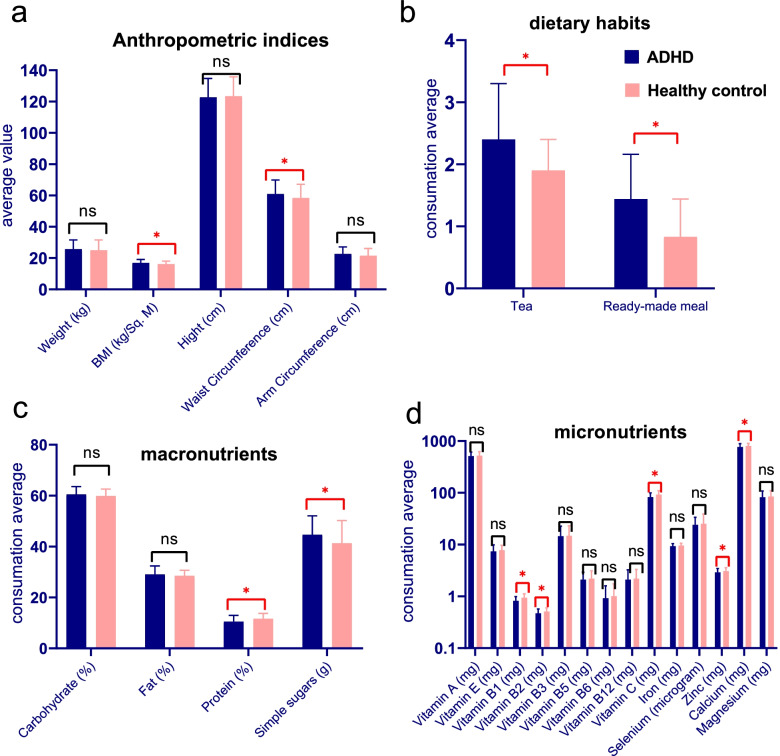
The mean values of anthropometric variables (**a**), dietary habits (**b**), macronutrients (**c**) and micronutrients (**d**) in ADHD children compared to healthy controls. Error bars represent standard deviation. ns = nonsignificant; asterisk = significant difference between groups

Table 3 and Fig. 1 compares the dietary consumption of macronutrients and micronutrients as well as some dietary habits between the case and control groups. Table 3 indicates that the children in the ADHD group significantly consume more simple sugars (*p* = 0.007), tea (*p* = 0.006), and Ready-made meals (*p* = 0.002), and lower vitamins B1, B2, C, Zn and calcium than those in the control group.

**Table 3:** Comparison of cronutrients as well as some dietary habits in case and control groups

Food	Case group	Control group	***P***-value
**Carbohydrate (energy percentage)**	60.48 ± 3.05	59.86 ± 2.71	NS
**Fat (energy percentage)**	29.03 ± 3.33	28.5 ± 2.15	NS
**Protein (energy percentage)**	10.49 ± 2.47	11.64 ± 2.12	0.001 *
**Simple sugars (g)**	44.66 ± 7.41	41.3 ± 8.93	0.007 *
**Tea (glasses)**	2.4 ± 0.9	1.9 ± 0.5	0.006 *
**Ready-made meal (weekly turn)**	1.44 ± 0.72	0.83 ± 0.61	0.002 *
**Vitamin A (mg)**	512.1 ± 100.1	524.23 ± 98.7	NS
**Vitamin E (mg)**	7.4 ± 2.5	7.8 ± 1.9	NS
**Vitamin B1 (mg)**	0.82 ± 0.17	0.94 ± 0.18	0.001 *
**Vitamin B2 (mg)**	0.47 ± 0.1	0.51 ± 0.09	0.004 *
**Vitamin B3 (mg)**	14.53 ± 8.1	14.8 ± 8.3	NS
**Vitamin B5 (mg)**	2.1 ± 0.82	2.2 ± 0.91	NS
**Vitamin B6 (mg)**	0.92 ± 0.68	1.01 ± 0.73	NS
**Vitamin B12 (mg)**	2.1 ± 1.15	2.2 ± 1.12	NS
**Vitamin C (mg)**	82.43 ± 17.51	92.26 ± 17.52	0.001 *
**Iron (mg)**	9.32 ± 1.1	9.6 ± 0.99	NS
**Selenium (micrograms)**	24.05 ± 9.6	25.19 ± 13.8	NS
**Zinc (mg)**	2.91 ± 0.55	3.06 ± 0.51	0.044*
**Calcium (mg)**	769.9 ± 121.03	803.82 ± 96.73	0.030*
**Magnesium (mg)**	82.05 ± 26.34	85.18 ± 25.09	NS

* T-test is statistically significant at *p* < 0.5

## Discussion

In this age and sex-matched case-control study, we compared nutrient intake, dietary patterns, and anthropometric variables of children with ADHD in comparison to healthy controls. The main findings of this study were lower levels of protein intake, higher poor dietary habits (e.g., consumption of ready-to-eat food), and lower levels of micronutrient intake such as vitamin C, vitamin B1, vitamin B2, calcium, zinc, and iron in children with ADHD compared to typically-developing peers. ADHD children had greater BMI values as well.

With regard to nutrient intake and dietary patterns, although there was no significant difference in total intake of carbohydrates and fat between the groups, consumption of simple sugars and tea was significantly higher and protein intake was significantly lower in children with ADHD. In the study by Ríos-Hernández et al. (2017), (mean age: 9.3 ± 2.8 years; 56.7% boys) there was no significant difference in dietary caloric intake and consumption of carbohydrate and fat between children with ADHD and normal children [21]. However, the level of simple sugars and caffeine consumption in children with ADHD was significantly higher. They also found that children with ADHD received less protein than healthy controls. It should be noted that although in their study consumption of micronutrients such as iron and zinc in the ADHD group was lower than the children in the control group, this difference was not statistically significant [21]. Furthermore, in a study by Azad Bakht et al. (2012) was found that in children with ADHD (mean age: 7 ± 2 years; 71% boys), carbohydrate intake is more than normal children. Also, vitamin C, vitamin B1, vitamin B2, calcium, zinc, and iron intakes in children with ADHD were significantly lower than healthy children; this finding is in line with our results [22]. Another relevant study in children with ADHD (mean age: 8.42 ± 1.72 years; 83.8% boys) found a series of negative correlations between ADHD symptoms and seafood and meat consumption (*p* = 0.006), dietary intake of zinc, protein, phosphorus, selenium, calcium, and riboflavin (*p* = 0.014), and the serum zinc level was negatively associated to ADHD (*p* = 0.003) [23].

In anthropometric indices, a non-significant difference was found between the arm circumference of ADHD children and normal children [24]. Another cohort study found that ADHD is not recognized a risk factor for significant weight gain from its normal level [25]. In our study, however, the arm circumference of children with ADHD was lower than normal children but this difference was not statistically significant. Different dietary patterns and socio-demographic or genetic factors may explain our novel results. In our study, in the ADHD group weight was slightly greater and height was slightly shorter compared to the control group. This was why the BMI was significantly greater in the ADHD group. Also, the abdominal circumference was significantly higher in the ADHD group. In line with our results, another study showed that the abdominal circumference and body fat percentage of non-medicated boys with ADHD was higher but the height was shorter than normal peers [13]. Along with what we found in the present study, the mean height of ADHD children was shorter compared to the control group in the study performed by Ríos Hernández et al. (2017) (136.5 ± 16.8 cm vs. 138.6 ± 17.3 cm, respectively). Also, the mean weight of children in the case group (ADHD) in their study was higher than the control group (38.1 ± 16.2 kg vs. 36.4 ± 14.5 kg; respectively) and there were no statistically significant differences between the two groups in these regards [21].

When it comes to height, results are also mixed. A study found that the height of non-medicated children with ADHD was significantly higher than normal children but after the beginning of pharmacotherapy, the growth velocity reduced so that those who remained on stimulant medication showed an annual growth rate about 20% less than what expected [26]. Moreover, in the two separate studies conducted by Ptacek et al. group, short height for age and sex was observed in non-medicated children with ADHD [13, 14]. In the present study, although the height of children with ADHD was slightly lower than the control group, however, the difference was not significant even after controlling for the impact of ADHD subtype and disorder severity. There is no significant difference in height at an earlier age compared to normal peers, however, over time, due to the growth retardation, the height of ADHD children remains shorter than normal children.

In this line, there is still controversy in various studies over the BMI difference between children with ADHD and their normal peers. In a relevant study, the BMI of children with ADHD was 19.6 ± 3.4 while it was 18 ± 3.3 in normal peers. Statistical analysis indicated that this difference was statistically significant [21]. Contrary to the above, the Mustillo et al. (2003) indicated no significant difference in BMI between children with ADHD and normal children [25]. In our study, in line with most studies, children with ADHD were more likely to have upper BMI than normal children. With respect to waist circumference, a study showed that children with ADHD who received no medication had significantly higher waist circumference than normal children [13]. On the other hand, the same researcher showed a significant difference in growth between children with ADHD that did not receive any medications and normal children when the sample size was doubled [14]. The synthesis of the results of this work by the researcher reveals that the involvement of some anthropometric indices, and reduction in growth velocity in children with ADHD, can be one of the clinical manifestations of the disease itself and not due to the complications of medications. ADHD children have low self-control so they show less food abstinence when it comes to food. Especially in the consumption of fast food and high in sugar. This reduces their balance, weight, and height.

Finally, we observed differences in eating behavior between ADHD children and healthy controls. Children with ADHD had a significantly higher waist circumference which is an indicator of fat tissue accumulation in the abdomen. Weight gain and fat accumulation in ADHD may be due to the association between impulsive behavior and loss of control in eating behaviors [27]. Furthermore, it is known that aggression and lack of attention in these children increase their appetite for food [28]. Besides, improper food patterns lead to an increase in body weight. According to the studies performed on food patterns in children with ADHD, the results indicated a high consumption of simple sugars, ready-made meals and a high-carbohydrate diet [29]. Due to the low levels of dopamine neurotransmitters, serotonin, and norepinephrine in the brain that lead to mood disorders and reduced desire to engage in physical activity, these children and adolescents are prone to overweight and obesity [30]. In children with ADHD, moving skills, executive functioning, and physical fitness are significantly lower compared to normal children who may contribute to obesity [31]. Overall, the results of this study indicate that there is a need to intervene in the lifestyle and eating behavior of children with ADHD. One interesting point here is the potential link between poor nutritional behavior and sleep problems in ADHD. It is known that there is an association between micronutrient status including some of those that ADHD children had lower levels (e.g., Zink, magnesium, iron) and sleep patterns [32]. On the other hand, ADHD comes with sleep difficulties and circadian problems [33] which are easily affected by lifestyle changes including the pandemic situation [34, 35]. Life style interventions should thus include not only eating behavior but other related factors such as sleep pattern.

Some limitations should be considered in our study. Despite the instructions and guidelines delivered to parents, studied food pattern may have missed some items, including snacks used in schools, which may children provide from school buffet. Other limitations of this study include the small sample size. Furthermore, parents’ awareness of ADHD may affect the children’s dietary patterns. It is suggested that Martin’s anthropometric measuring instruments be used for weight measurement in future research.

## Conclusion

Children with ADHD have higher waist circumference and BMI than healthy children, and this difference is related to the severity of the disease and its type. The BMI and waist circumference in children with mild ADHD and mostly attention-deficit type was significantly higher than the control group. Children with ADHD consume more simple sugars, tea, and ready-made foods but lower protein, vitamin B1, vitamin B2, vitamin C, zinc and calcium compared with their normal control peers. These results suggest lifestyle and nutritional interventions in children with ADHD as early as possible.

## Data Availability

The datasets used and/or analyzed during the current study are available from the corresponding author on reasonable request.

## Abbreviations

ADHD: Attention-deficit and Hyperactivity Disorder
FFQ: Food Frequently Questionnaires
BMI: Body Massive Index
DSM-5: Diagnostic and Statistical Manual of Mental Disorders
